# AUTOSURV: INTERPRETABLE DEEP LEARNING FRAMEWORK FOR CANCER SURVIVAL ANALYSIS INCORPORATING CLINICAL AND MULTI-OMICS DATA

**DOI:** 10.21203/rs.3.rs-2486756/v1

**Published:** 2023-08-08

**Authors:** Lindong Jiang, Chao Xu, Yuntong Bai, Anqi Liu, Yun Gong, Yu-Ping Wang, Hong-Wen Deng

**Affiliations:** 1. Tulane Center of Biomedical Informatics and Genomics, School of Medicine, Tulane University, New Orleans, LA, 70112; 2. Department of Biostatistics and Epidemiology, University of Oklahoma Health Sciences Center, Oklahoma City, OK, 73104; 3. Department of Biomedical Engineering, School of Science and Engineering, Tulane University, New Orleans, LA, 70118

**Keywords:** Deep learning, multi-omics integration, survival analysis, breast cancer, ovarian cancer

## Abstract

Accurate prognosis for cancer patients can provide critical information for optimizing treatment plans and improving life quality. Combining omics data and demographic/clinical information can offer a more comprehensive view of cancer prognosis than using omics or clinical data alone and can reveal the underlying disease mechanisms at the molecular level. In this study, we developed a novel deep learning framework to extract information from high-dimensional gene expression and miRNA expression data and conduct prognosis prediction for breast cancer and ovarian cancer patients. Our model achieved significantly better prognosis prediction than the conventional Cox Proportional Hazard model and other competitive deep learning approaches in various settings. Moreover, an interpretation approach was applied to tackle the “black-box” nature of deep neural networks and we identified features (i.e., genes, miRNA, demographic/clinical variables) that made important contributions to distinguishing predicted high- and low-risk patients. The identified associations were partially supported by previous studies.

## Introduction

Cancer is one of the leading causes of death worldwide [1]. It is estimated that in 2022, 1,918,030 new cases will be diagnosed, and about 609,360 people will die from cancer (i.e., almost 1700 deaths per day) in the United States [2]. Accurate cancer prognosis prediction helps clinicians to conduct more appropriate treatment allocation for patients to prolong life span, increase life quality, and reduce unnecessary treatment cost. Recent studies have applied machine learning (ML) techniques in the analysis of clinical and genomic features, and they showed that ML has improved performance in cancer susceptibility, recurrence, and survival prediction compared to traditional approaches (e.g., Kaplan-Meier method) [3–5]. In practice, several issues can undermine the credibility of model predictions. Firstly, measuring some important clinical variables (e.g., disease stage) relies heavily on the clinician’s individual interpretation, which may introduce human bias, thereby reducing the accuracy and reliability of the prediction results [1]. Interestingly, the study [5] showed that ML model can give more accurate predictions than the treating physicians in cancer survival analysis. Secondly, small sample size accompanied by high-dimensional input data (e.g., gene expression data, whole slide image, etc.) can result in overfitting [6] and hamper the generalizability of existing models. Thirdly, the relationship between predictors and survival outcome may be non-linear [7], and thus existing models that assume a linear relationship (e.g., Cox Proportional Hazards model [8]) can produce inaccurate results.

Following the widespread application of high-throughput sequencing technologies, omics data (e.g., mRNA expression data, miRNA expression data) have become more accessible than ever. Incorporating omics information in analyses could provide models with a more comprehensive view and mitigate the bias brought by a single data type. Furthermore, this could help us understand disease mechanisms at the molecular level. Some recent studies included omics information in their models for cancer classification or prognosis [1, 6, 9–15]; moreover, several studies [11, 13–15] have shown that integrating multi-omics data can improve model performance compared with single-omics. Thus, efficient, and effective incorporation of multi-omics data into cancer survival analysis is worth further investigation.

There are many approaches available for survival analysis, such as the Cox proportional hazard (CoxPH) model and some newly developed Deep Neural Networks (DNN) [1, 6, 9–12, 14, 16]. CoxPH model assumes a linear relationship between a patient’s log-risk of failure and covariates [16, 17]. Although it is commonly applied for survival analysis, the CoxPH model cannot handle complex data structure well. In addition, due to the high-dimension-low-sample-size issue [6], directly applying CoxPH on omics data can cause overfit.

On the other hand, DNN can deal with non-linear relationships intrinsically, which can well represent complicated data structures. By virtue of their flexibility, deep learning (DL) models can be designed (or combined with other approaches) to conduct feature extraction and integration from high-dimensional omics data. DeepSURV [16] is a model that is based on CoxPH but adopts a DNN structure. Katzman et al. demonstrated that DeepSURV was able to outperform the CoxPH model in prognosis prediction under various scenarios, highlighting the strength of deep learning models in handling complex data patterns compared to conventional approaches. In a similar fashion, Cox-nnet [1] and CoxPASNet [6] both adopted feed-forward DNN structures for prognosis prediction, but unlike DeepSURV, they can handle high-dimensional gene expression data. Moreover, CoxPASNet applies a sparse coding technique to further reduce the risk of overfitting. In the study [6] CoxPASNet showed significantly better performance than Cox-nnet. OmiVAE is a DNN that combines gene expression and DNA methylation data for cancer classification [15]. OmiVAE consists of a variational autoencoder (VAE) and a downstream classification network. It can achieve task-oriented feature extraction and patient classification simultaneously in the supervised phase of its training scheme [15]. Zhang et al. showed that OmiVAE performed better when trained from multi-omics data than single-omics data. SALMON [11] implemented local maximal Quasi-Clique Merger (lmQCM) [18] for co-expression network analysis. The first principal components of the identified gene/miRNA co-expression modules were then extracted and input into a CoxPH Regression Network [11]. In their study, Huang et al. observed improved performance of SALMON when more omics data were incorporated. Cheerla et al. built a DL model that integrates multimodal representations from clinical, omics, and whole slide image data and performs pan-cancer prognosis prediction [10]. MultiSurv, proposed by [12], is another DL-based pan-cancer prognosis prediction model. It also takes those three modalities as the input. But it applies a different integration approach (i.e., computing the row-wise maximum of the feature representation matrix) and does not depend on the proportional hazard assumption of the CoxPH model (i.e., owing to their implementation of the discrete-time survival model formulation). Finally, for result interpretation and feature importance investigation, previous DL approaches have applied gradient-based or perturbation-based methods [6, 11, 13].

In this paper, we introduce a novel deep learning model for prognosis prediction, namely, AUTOSurv. This model uses multi-omics data and tackles the high-dimension-low-sample-size issue through dimension reduction. We demonstrate that by virtue of its special structure design and learning strategy, AUTOSurv obtained significantly better prognosis prediction performance compared to other existing deep learning methods in various cases. We also revealed the strength and weakness of end-to-end task-oriented feature extraction strategy implemented in OmiVAE and observed that VAE models can be more effective at dimension reduction than WGCNA [19] plus principal component analysis (PCA) under certain conditions. Finally, to resolve the “black-box” nature of DNNs, we applied the DeepSHAP interpretation approach [20–22] to the learned AUTOSurv model and identified important genes, miRNA and pathways that contributed to distinguishing between high- and low-risk patients. To our knowledge, this is the first time that an activation-based interpretation approach was applied to a survival analysis-related deep neural network. Together, we hope our work could be a first step towards the development of more advanced deep learning approaches that not only can provide accurate prognosis prediction but also can unravel hidden mechanisms underlying cancer survival.

## Results

### Performance of AUTOSurv using different types of omics data and data integration methods.

We applied AUTOSurv to both The Cancer Genome Atlas (TCGA) Breast (BRCA) and Ovarian (OV) cancer datasets (see Methods section for details about data collection and preprocessing), and four different cases were designed to evaluate its performance. In Case 1, gene expression data, miRNA expression data, and demographic/clinical data (e.g., age, disease stage, race) were used as model input. In this case, the KL-PMVAE section of AUTOSurv combines gene expression and miRNA expression information (with gene expression information encoded in the pathway nodes) to derive a joint set of latent features for the two omics data types (although mRNA and miRNA expression data both come from transcriptome, here we treat them as two omics because gene regulation by miRNA is part of epigenetic mechanisms [23]), as illustrated in Figure 6b. We denote this omics integration strategy as the “entangle” approach. The LFSurv section of AUTOSurv then takes this joint set of latent features and the demographic/clinical data as input and calculates prognostic index (*PI*) for each patient where higher PI implies higher risk of death, as illustrated in Figure 6c. In Case 2 and Case 3, demographic/clinical data, and a single type of omics data (i.e., gene expression and demographic/clinical data for Case 2; miRNA expression and demographic/clinical data for Case 3) were used as model input. Modified KL-PMVAE networks extract latent features $\mu_{gene}$ and $\mu_{miRNA}$ from the two types of omics data respectively, as illustrated in Supplementary Figure 2a-b. LFSurv takes either $\mu_{gene}$ or $\mu_{miRNA}$ (plus demographic/clinical data) as input, as illustrated in Supplementary Figure 2c. In Case 4, gene expression, miRNA expression, and demographic/clinical data were used as model input again, but this time LFSurv takes the direct concatenation of $\mu_{gene}$ and $\mu_{miRNA}$, instead of a joint set of latent features as input. We denote this omics integration strategy as the “concatenate” approach and illustrate this in Supplementary Figure 2c. C-index was used to measure model performance; it values between 0 and 1 and higher C-index indicates more accurate prognosis prediction (see Methods section for more detail).

**Figure 1:**
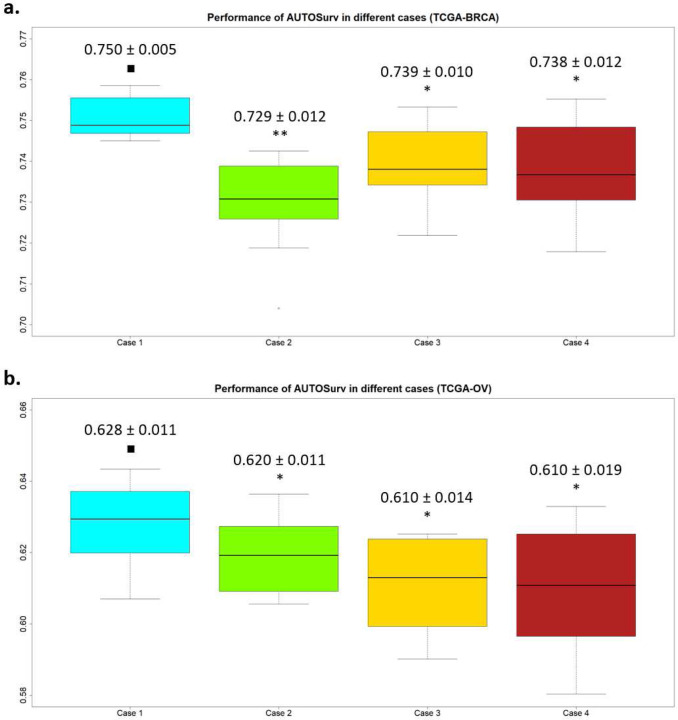
Performance of AUTOSurv in terms of testing set C-index. The prediction performance of AUTOSurv was evaluated on two datasets (i.e., TCGA-BRCA (a) and TCGA-OV (b)) in four different cases: **Case 1:** Original KL-PMVAE model (see Methods section) implemented where “entangle” omics integration approach was applied; gene expression data, miRNA expression data, and demographic/clinical data (e.g., age, disease stage, race) were used as model input; **Case 2:** Gene expression and demographic/clinical data were used as model input. KL-PMVAE was modified according to Supplementary Figure 2a; **Case 3:** MiRNA expression and demographic/clinical data were used as model input. KL-PMVAE was modified according to Supplementary Figure 2b; **Case 4:** “Concatenate” omics integration approach (Supplementary Figure 2c) was applied; gene expression, miRNA expression, and demographic/clinical data were used as model input. One-tailed Wilcoxon signed-rank tests were conducted to compare performance between Case 1 and Cases 2, 3, and 4, respectively. ∎: baseline model; *: P-value < 0.05; **: P-value < 0.005. Y-axis: C-index.

As the results demonstrated in Figure 1, it is obvious that compared to the “concatenate” approach in Case 4, the “entangle” approach in Case 1 had superior performance in integrating the two types of omics data for prognosis prediction in terms of C-index (i.e., for TCGA-BRCA: 0.750 ± 0.005 vs 0.738 ± 0.012, one-tailed Wilcoxon signed-rank test p-value = 0.005; for TCGA-OV: 0.628 ± 0.011 vs 0.610 ± 0.019, one-tailed Wilcoxon signed-rank test p-value = 0.019). Interestingly, for both datasets, compared to Case 2 and Case 3 where only a single type of omics data was considered, directly concatenating latent features from two omics data types in Case 4 did not render better prediction performance (one-tailed Wilcoxon signed-rank test p-value ≥ 0.1 for all comparisons). One possible explanation is that given that miRNA mostly affects the phenotype by regulating the expression of certain genes, it is likely that the survival-related-information underlying gene expression and miRNA expression data, respectively, have some degree of overlap. Therefore, if concatenated directly, the presumably overlapped information in $\mu_{gene}$ and $\mu_{miRNA}$ can be redundant for LFSurv to predict prognosis. Hence, no significant improvement was observed compared to using only $\mu_{gene}$ or $\mu_{miRNA}$ (Case 2 or Case 3). On the other hand, when the “entangle” integration approach was applied as in the original KL-PMVAE (Case 1), the decoder of KL-PMVAE was trained to reconstruct the two types of omics data simultaneously from a common set of latent features (see Methods section). The aforementioned information overlap might facilitate a crosstalk between the two omics data types and help to extract the most relevant information for the reconstruction task. Thus KL-PMVAE in Case 1 achieved more efficient feature extraction than single-omics VAE models in Case 2 and Case 3.

To assess the influence of omics data (i.e., gene expression and miRNA expression data) on prognosis prediction, we fitted LFSurv using only demographic/clinical variables and obtained testing set C-index 0.713 ± 0.008 for TCGA-BRCA dataset and 0.617 ± 0.015 for TCGA-OV dataset. One-tailed Wilcoxon signed-rank test gave p-value < 0.001 for TCGA-BRCA dataset and p-value = 0.07 for TCGA-OV dataset compared to Case 1 of AUTOSurv (C-index = 0.750 ± 0.005 for TCGA-BRCA; C-index = 0.628 ± 0.011 for TCGA-OV). It suggests that incorporating omics information improved prediction performance of AUTOSurv, and this improvement was greater for the TCGA-BRCA dataset. This finding also implied that the amount of survival-related information embedded in omics data might vary across different cancer types, and this may raise the issue of optimizing resource distribution in data collection process to focus on more informative omics types and conduct more cost-effective survival analysis/prediction for different cancer types. Nevertheless, considering the noticeable gap in sample size between the TCGA-BRCA and TCGA-OV datasets (i.e., 1058 vs 355), this statement may require further verification, which is out of the scope of this study.

We compared prognosis prediction performance of LFSurv network with the conventional CoxPH model using only demographic/clinical data. We trained a CoxPH model using the whole tuning set and applied the trained model to the testing set (see Methods section for more details about data division); testing set C-index of 0.673 (one-tailed Wilcoxon signed-rank test p-value < 0.001 vs LFSurv) and 0.606 (one-tailed Wilcoxon signed-rank test p-value = 0.02 vs LFSurv) were obtained for TCGA-BRCA dataset and TCGA-OV dataset respectively (because no hyperparameter was applied for the CoxPH model, in each dataset we only have to train the model once obtaining one testing set C-index). This implied that some higher-order interactions between the demographic/clinical variables captured by the hidden layer of LFSurv are potentially important for survival analysis. It showed the strength of deep neural networks in utilizing more complex data structure/feature relationships compared to the conventional CoxPH model.

### Performance of AUTOSurv compared to other deep learning approaches.

**Figure 2:**
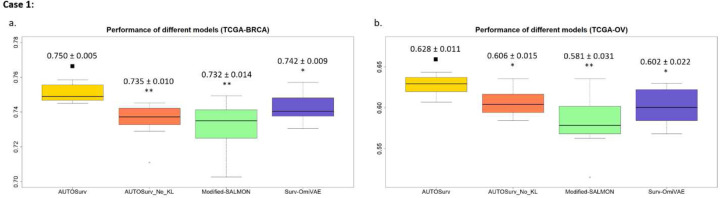

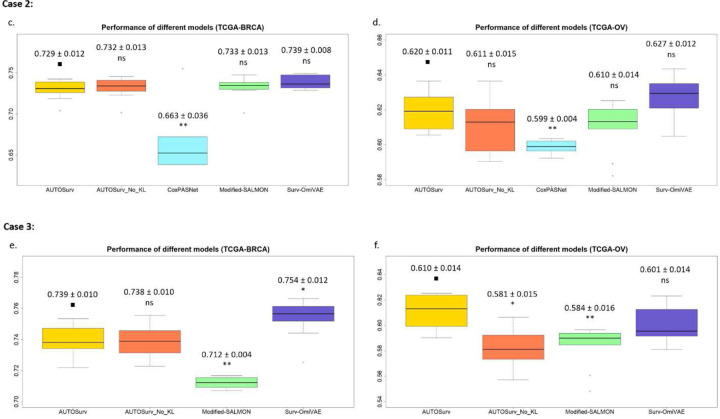
Comparison between different models in terms of testing set C-index. Prediction performance of the models were evaluated on two datasets (TCGA-BRCA (a, c, e) and TCGA-OV (b, d, f)) in three different cases: **Case 1:** Gene expression data, miRNA expression data, demographic/clinical information all included as input. For AUTOSurv, original KL-PMVAE with “entangle” omics integration approach was applied; **Case 2:** Gene expression data and demographic/clinical information included as input; **Case 3:** MiRNA expression data and demographic/clinical information included as input. One-tailed Wilcoxon signed-rank tests were conducted to compare performance between AUTOSurv and other DL approaches (including AUTOSurv without KL-annealing) in the same cases. ∎: baseline model; *: P-value < 0.05; **: P-value < 0.005; ns: not significant. AUTOSurv_No_KL: AUTOSurv without KL-annealing. Y-axis: C-index.

We fitted other recently developed and representative deep learning models (CoxPASNet [6], OmiVAE [15], and SALMON [11]) using the same settings of Case 1, Case 2, and Case 3 as mentioned earlier and compared their performance with AUTOSurv. The results are summarized in Figure 2.

CoxPASNet was not originally designed to handle multi-omics data, so we used only gene expression data and demographic/clinical variables for this model. The testing set C-index for CoxPASNet was 0.663 ± 0.036 for the TCGA-BRCA dataset and 0.599 ± 0.004 for the TCGA-OV dataset, which was significantly lower than that obtained by AUTOSurv (TCGA-BRCA: C-index 0.729 ± 0.012, p-value = 0.002; TCGA-OV: C-index 0.620 ± 0.011, p-value = 0.003) using the same input data. This implied that dropout combined with sparse coding might not be efficient enough when dealing with high-dimensional omics features in an end-to-end feed-forward deep neural network.

As mentioned in the Introduction, OmiVAE is another end-to-end deep learning model. Here we tailored OmiVAE to survival analysis as Surv-OmiVAE, which connects the encoder of KL-PMVAE to LFSurv and trains them together to achieve “task-oriented feature extraction” in its supervised phase [15]. From Figure 2 we see that Surv-OmiVAE achieved high performance in single-omics cases (i.e., Case 2 of TCGA-OV; Case 3 of TCGA-BRCA). In Case 3 of TCGA-BRCA dataset, Surv-OmiVAE outperformed AUTOSurv significantly. When multi-omics data were considered, however, Surv-OmiVAE did not gain improvement in performance from the extra omics information. This suggests that “task-oriented feature extraction” can potentially help capture survival-related information in the latent features and hence increase the prediction accuracy of LFSurv. Nevertheless, adaptations are needed to accommodate multi-omics scenarios and make full use of information from different omics-types.

For our implementation of SALMON, the widely applied WGCNA approach [19] was adopted for co-expression network analysis. The first principal components of the identified gene/miRNA co-expression modules were taken as eigengenes/eigen-miRNAs and input into LFSurv for prognosis prediction. Figure 2 shows that AUTOSurv achieved comparable or better performance compared to the modified-SALMON when single omics data were used as input in Case 2 and Case 3. This implied that VAE could be more powerful than “WGCNA plus PCA” in dimension reduction for certain types of expression data (i.e., miRNA expression data). In Case 1 of modified-SALMON, the eigengenes and eigen-miRNAs were concatenated and fed to LFSurv; not surprisingly, the performance did not improve compared to Case 2 where only eigengenes were incorporated (one-tailed Wilcoxon signed-rank test p-value = 0.246 for TCGA-BRCA; p-value = 0.009 for TCGA-OV). This could be due to the same information overlap between the two omics data types mentioned above.

Finally, in Case 1 when both types of omics data and demographic/clinical variables were used as input, AUTOSurv outperformed other approaches significantly (including AUTOSurv without KL-annealing) for both datasets (Figure 2a–b).

### Performance of AUTOSurv with and without KL-annealing

We tested the utilization of KL-annealing learning strategy (see Methods section) in AUTOSurv. When only single-omics data were used (Figure 2 Case 2, Case 3), the prediction performance of AUTOSurv with and without KL-annealing did not differ significantly (except for Case 3 of TCGA-OV dataset, where AUTOSurv with KL-annealing achieved significantly better performance, one tailed Wilcoxon signed-rank test p-value = 0.007). When two omics data types were modelled simultaneously as in Case 1, however, the performance of AUTOSurv with KL-annealing was significantly better (p-value = 0.003 for TCGA-BRCA dataset; p-value = 0.007 for TCGA-OV dataset). This implies that KL-annealing helped retain useful information in the latent features when the reconstruction task of KL-PMVAE became more complicated. Moreover, for AUTOSurv without KL-annealing, Case 1 performance did not improve compared to Case 2 or Case 3 (except for Case 1 vs Case 3 of TCGA-OV, p-value equals 0.005). Therefore, it is reasonable to assume that the combination of the “entangle” omics integration approach and KL-annealing, instead of the “entangle” approach alone, made AUTOSurv more efficient than other DL models when both omics data types were incorporated. These findings highlight the subtlety in selecting a plausible structure and optimization strategy when constructing deep neural networks. Moreover, they encourage us to explore in future studies whether KL-annealing has the potential to boost the performance of VAE models in more complex integration tasks involving more than two types of omics data.

### Risk group assignment and DeepSHAP interpretation

The AUTOSurv models that yielded the highest testing C-index in Case 1 (Figure 1 a and b for TCGA-BRCA dataset and TCGA-OV dataset, respectively) were used as the final models to predict patients’ risk levels and identify important features via DeepSHAP.

**Figure 3:**
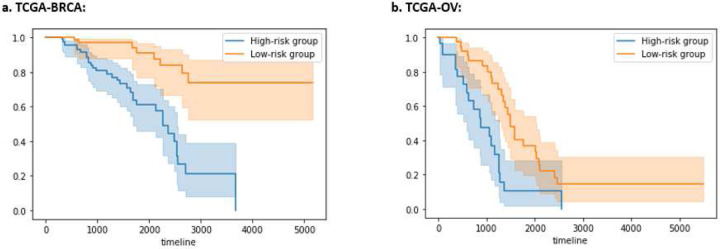
Kaplan-Meier curves for the high-risk and low-risk patient groups in the testing set for (a) TCGA-BRCA dataset, Log-rank test returned test statistic 23.09 (p-value < 0.005); (b) TCGA-OV dataset, Log-rank test returned test statistic 12.20 (p-value < 0.005). Unit of X-axis: days. Y-axis: proportion of surviving patients. Shaded area: 95% confidence interval for the survivor function.

For each dataset (i.e., TCGA-BRCA or TCGA-OV), after training on the tuning set, we saved the AUTOSurv model and the median prognostic index ($PI_{med}$) in the tuning set patients. We applied AUTOSurv to predict new patients’ risk levels using the testing set. Patients having predicted $PI\;>\;PI_{med}$ were assigned to the high-risk group. Otherwise, they were assigned to the low-risk group. For both datasets, Figure 3 shows highly significant differences between Kaplan-Meier curves of the two predicted risk groups (Log-rank test p-value < 0.005).

We conducted an interpretation study via DeepSHAP (see Methods section) based on tuning set data, since AUTOSurv was trained on this dataset and achieved good prediction performance on the testing set (i.e., highest C-index 0.759 for TCGA-BRCA dataset; highest C-index 0.6434 for TCGA-OV dataset), which implies that the interpretation results on tuning set should be generalizable. Table 1 summarizes the top 10 LFSurv input features with the highest overall contribution scores when explaining the difference in predicted PI values between high- and low-risk groups (overall contribution scores for all LFSurv input features can be found on our Github website: https://github.com/jianglindong93/AUTOSurv).

From Table 1 we see that for both datasets, features with the highest overall contribution scores are clinical variables (age and clinical stage in the TCGA-BRCA dataset together accounted for 46.81% of the total sum of overall contribution scores; age and race in the TCGA-OV dataset together accounted for 45.52% of the total sum of overall contribution scores). This shows that clinical variables can play important roles in survival analysis and should not be ignored even when omics data are available.

**Figure 4:**
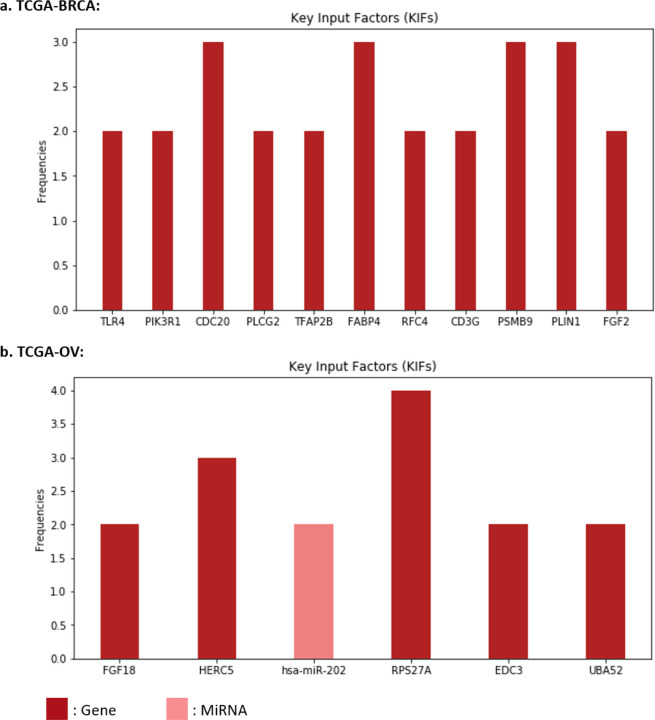
Identified Key Input Factors (KIFs). X axis shows the names of input factors corresponding to (a) 11 genes for the TCGA-BRCA dataset, and (b) 5 genes and 1 miRNA for the TCGA-OV dataset. Y axis shows the frequency for each gene/miRNA, that is, the number of latent features that included this gene/miRNA as one of their top 10 input factors that contributed most to their value differences between high- and low-risk groups.

For each of the datasets, a total of 16 latent features (i.e., {*μi*}*i*=1,…,16) were extracted from the two omics data types and input into LFSurv. The number of latent features to extract (i.e., number of latent features in the bottleneck layer) was tuned as a hyperparameter, and 16 was chosen because it was in the best set of hyperparameters that gave smallest reconstruction loss for KL-PMVAE (for both datasets). The numbers of latent features we tuned across were summarized in Supplementary Table 3.1. To identify pathways/genes/miRNAs that contributed most to the important latent features, we calculated KL-PMVAE input factor (gene/miRNA) contribution scores for each of the top 6 latent features in Table 1 (i.e., *μ*5, *μ*2, *μ*10, *μ*8, *μ*1, *μ*9 for TCGA-BRCA; *μ*16, *μ*3, *μ*4, *μ*10, *μ*12, *μ*6 for TCGA-OV). For each dataset, if a gene or miRNA had high contribution scores (i.e., top 10 among all input factors) for more than one latent feature, we identified it as a Key Input Factor (KIF). In Figure 4, we present the identified KIFs for TCGA-BRCA and TCGA-OV with their frequencies as top 10 most contributing factors of latent features.

For TCGA-BRCA dataset, the identified KIFs included 11 genes, all of which were found to be associated with breast cancer in existing studies [24–34]. For example, among the 4 genes with frequency 3 in Figure 4a: CDC20, FABP4, PSMB9, and PLIN1, the study [24] found that CDC20 knockdown inhibited the migration of metastatic MDA-MB-231 breast cancer cell line. Recent studies also demonstrated that FABP4 promotes obesity-associated breast cancer development [32]. For PSMB9, the study [28] found that PSMB9 was overexpressed in breast cancer cells. For PLIN1, the study [33] found that its mRNA expression is significantly downregulated in human breast cancer.

For TCGA-OV dataset, we identified 5 genes and 1 miRNA as KIFs (Figure 4b). Four of these factors (FGF18, HERC5, hsa-miR-202, and RPS27A) were found to be associated with ovarian cancer in previous literatures [35–38]. Overexpression of FGF18 was identified as a predictive marker for poor clinical outcomes in patients with advanced stage, high-grade serous ovarian cancer by [35]. HERC5 was found to have increased expression levels in topotecan-resistant ovarian cancer cell lines by [36]. For hsa-miR-202, [37] found that miR-202-5p was down-regulated in ovarian cancer and verified the role of miR-202-5p in suppressing cell proliferation, migration, and invasion in ovarian cancer. The study [38] identified genes with survival-related alternative splicing events in ovarian cancer, and RPS27A was one of the hub genes in the gene interaction network.

**Figure 5:**
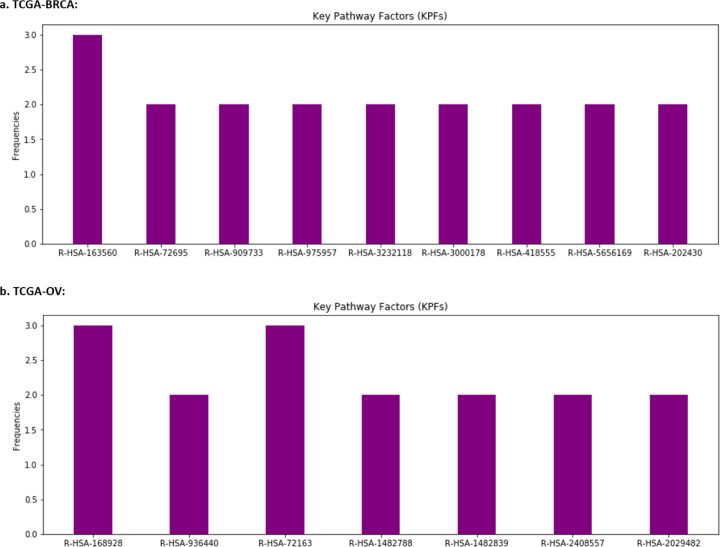
Identified Key Pathway Factors (KPFs). X axis shows the names of those pathways in (a) TCGA-BRCA dataset and (b) TCGA-OV dataset. Y axis shows the frequency for each pathway, that is, the number of latent features that included this pathway as one of the top 10 pathways that contributed most to their value differences between high- and low-risk groups.

We applied the same procedure to identify the Key Pathway Factors (KPFs) for the top 6 latent features. The identified pathways and their frequencies are illustrated in Figure 5. For TCGA-BRCA, evidence of association with breast cancer or cancer in general can be found for all the identified pathways in previous literatures [39–48]. The pathway with frequency three in Figure 5a, R-HSA-163560, is triglyceride catabolism. According to [46], triglyceride was found to be significantly elevated among breast cancer patients compared to controls, and their study suggested that higher levels of triglyceride may play important role in carcinogenesis. Using DeepSHAP, we were able to identify KIFs that contributed most to KPFs. For the KPF: R-HSA-163560 in the pathway layer, the top two contributing factors were KIFs: FABP4 and PLIN1. According to [49], FABP4 was positively associated with triglycerides in breast cancer patients. The study [33] noted that PLIN1 plays a distinct role in regulating both triglyceride storage and lipolysis in adipocytes, and that reduced expression of PLIN1 could be an independent predictor of overall survival for breast cancer patients.

For the TCGA-OV dataset, evidence of association with ovarian cancer can be found in previous literatures for six of the identified pathways (i.e., R-HSA-168928, R-HSA-936440, R-HSA-72163, R-HSA-1482788, R-HSA-1482839, R-HSA-2408557 [50–56]). The two pathways with frequency three in Figure 5b are R-HSA-168928 and R-HSA-72163, which correspond to RIG-I/MDA5 mediated induction of IFN-alpha/beta, and mRNA splicing, respectively. According to [52], it has been reported that IFN-alpha specifically targets a subset of ovarian cancer cells that have stem-like properties. The study [54] found that high expression of RIG-I is associated with poor clinical outcomes in ovarian cancer. The study [55] conducted prognosis prediction for ovarian cancer patients based on alternative splicing (AS) events and suggested AS sites as potential targets for ovarian cancer treatment. Pathway R-HSA-168928 includes three of the KIFs in Figure 4b (HERC5, RPS27A, and UBA52). For the node corresponding to R-HSA-168928 in the pathway layer of KL-PMVAE, HERC5 is the input factor that had the highest contribution score with respect to the difference in its node values between high- and low-risk groups.

Overall, the results suggest that DeepSHAP has the potential to reveal hidden mechanisms underlying breast and ovarian cancer prognosis and may provide support and guidance for future molecular-level investigations.

## Discussion

AUTOSurv is a deep learning model consisting of a specially designed upstream KL-PMVAE network that extracts lower-dimension latent features from high-dimensional omics data; and a downstream multi-layer perceptron LFSurv that receives the combined input of the extracted latent features and the demographic/clinical variables and calculates a predicted prognostic index (*PI*) for each patient. We applied AUTOSurv in different cases. AUTOSurv achieved the highest C-index when gene expression, miRNA expression, and clinical data were all used. Moreover, the highest C-index was achieved in the case where the “entangle” omics integration design was combined with the KL-annealing learning scheme. These results imply that although incorporating more types of data can potentially boost model performance, the method used to integrate different types of information determines whether the model has a more “comprehensive” view or a “noisier” view. Although the incorporation of omics data improved model performance (C-index increased from 0.713 ± 0.008 to 0.750 ± 0.005 for TCGA-BRCA dataset; C-index increased from 0.617 ± 0.015 to 0.628 ± 0.011 for TCGA-OV dataset), clinical variables (e.g., age, disease stage, race) were assigned the highest contribution scores by DeepSHAP among all input features of LFSurv. This suggests that clinical variables are vitally important for survival analysis and should not be ignored regardless of access to other types of modalities.

By applying DeepSHAP, we identified genes, miRNA, and pathways that were important for distinguishing predicted high- and low-risk group patients, most of which were found to be associated with breast/ovarian cancer or cancer in general in previous studies. This is reassuring as it implies that it is indeed the biologically relevant information rather than random events that is guiding the model predictions. By virtue of the interpretation-friendly design of KL-PMVAE, we linked the key pathways with the key genes. This showed that “AUTOSurv + DeepSHAP” could help us 1) identify potential biomarkers for cancer prognosis and 2) reveal which pathways will provide insight into hidden mechanisms. To our best knowledge, this is the first study to apply an activation-based interpretation method to a survival analysis-oriented DNN. Nonetheless, the procedure for selecting key features can be further improved and standardized to obtain more reliable and robust interpretations. For instance, we could utilize the Shapley values (i.e., contribution scores, see Methods section) generated by DeepSHAP as quantitative measures instead of focusing only on their rankings. We could also consider the +/− signs of the Shapley values to make our interpretation more informative. The way of incorporating those signs, however, need to be selected with caution so the positive and negative contribution scores won’t simply cancel each other out hence undermine the importance of the features.

In this study, we assumed that the overlapping information between gene expression and miRNA expression data facilitated more efficient multi-omics integration. The view-specific information (i.e., here we consider each type of omics data as a different view of the samples [13]), however, can also be important especially when the overlapping information between different types of omics data is trivial. Moreover, disentangling view-specific and view-shared aspects of latent features may make the VAE more interpretable. Models like Deep Probabilistic CCA (DPCCA) introduced by [57] might be useful for such disentanglement tasks and combining this approach with our KL-PMVAE for better multi-omics integration and feature extraction is a potential future direction of study. Integration of multiple modalities is another relevant topic, and some studies have attempted to include whole slide image data [10, 12] as one extra input modality for prognosis prediction. For AUTOSurv we concatenated the latent features (extracted from omics data) and the clinical variables directly in the input layer of LFSurv. This approach is straightforward, but our results suggest that concatenation may not be the best way to handle complex relationships between different modalities. We expect more delicate model designs to be developed for multimodal representation learning in our future pursuits.

There are several ways that AUTOSurv can be improved in the future. Firstly, although AUTOSurv achieved better performance in many cases compared to other DL models (i.e., CoxPASNet [6], OmiVAE [15], SALMON [11], with modifications made to better suit our purpose), in some cases, Surv-OmiVAE outperformed AUTOSurv. For example, in TCGA-BRCA dataset, when miRNA expression data and clinical variables were used as input, Surv-OmiVAE achieved C-index 0.754 ± 0.012 vs 0.739 ± 0.010 from AUTOSurv. This implies that proper modifications to AUTOSurv allowing task-oriented feature extraction may be a good starting point to developing a more advanced model. Secondly, in this study we excluded many genes to facilitate the “pathway-masking” design of KL-PMVAE (see Methods section for more details), which could cause information loss. Improvements in the pre-filtering process will be another focus in future studies. For instance, we could combine multiple pathway databases and/or change the selection criteria for the pathway nodes (e.g., only exclude pathways that contain fewer than 10 genes or greater than 500 genes in our dataset) to expand genome coverage. Finally, the downstream LFSurv section of AUTOSurv is a Cox Proportional Hazard network, and studies have tried to overcome the proportional hazard constraint to yield more realistic predictions [12, 14, 58]. We could also adjust the learning objectives of AUTOSurv accordingly to model time-varying effects of the input features and/or to learn patient-specific survival distributions [14, 58].

Finally, although we only studied breast cancer and ovarian cancer data in this paper, our approach can be directly implemented to perform prognosis prediction and result interpretation for other cancer types.

## Methods

### Overview of AUTOSurv.

**Figure 6:**
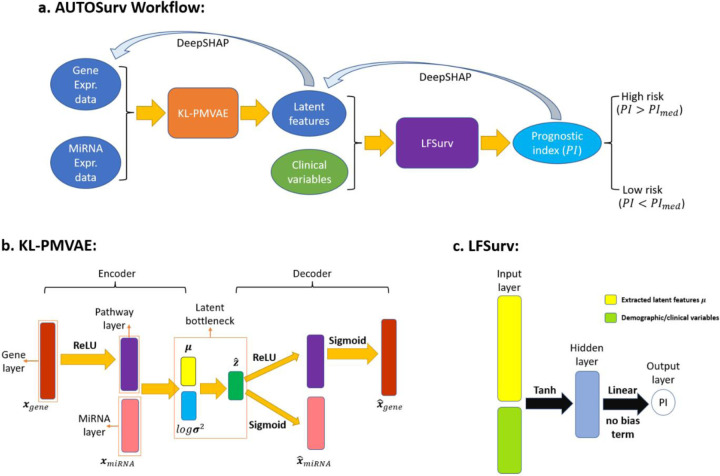
AUTOSurv workflow and key components illustration. (a) Workflow of AUTOSurv. KL-PMVAE was trained to conduct integration and dimension reduction on gene expression and miRNA expression data. Latent features generated by KL-PMVAE will be combined with the demographic/clinical variables and fed into the LFSurv network. The output of LFSurv will be a prognostic index (PI) for each patient that reflects the patient’s risk of death. DeepSHAP will be applied to locate important input features (e.g., genes) that contribute to the difference in predicted prognosis between high- and low-risk patient groups. PI : median prognostic index. (b) Illustration of KL-PMVAE. The VAE model consists of an encoder and a decoder. The encoder has one gene layer (each node represents a gene), one pathway layer (each node represents a pathway), and one miRNA layer (each node represents a miRNA) and learns a distribution estimate of the latent variables z (parameterized by means μ and variances $\sigma^{2}$ which were stored in the latent bottleneck). The decoder takes a sample $\hat{z}$ from the distribution estimate as input and outputs the reconstructed expression data ${\hat{x}}_{miRNA}$ and ${\hat{x}}_{gene}$. (c) Illustration of LFSurv. This network consists of an input layer, a hidden layer, and an output layer with only one node. The extracted latent features μ were concatenated with the demographic/clinical variables. The network receives the concatenated features and outputs the prognostic index (PI).

AUTOSurv is a deep learning model for cancer prognosis prediction combining multi-omics and demographic/clinical data. There are three major parts to our framework: (1) pathway information incorporated multi-omics variational autoencoder (VAE) with KL-annealing learning strategy [59] (KL-PMVAE) for efficient multi-omics integration and latent feature extraction; (2) latent-feature-fed survival network (LFSurv) integrates latent features extracted by the VAE model with demographic/clinical variables and conducts final prognosis prediction; and (3) DeepSHAP [21, 22] interpretation approach applied to the trained AUTOSurv model (KL-PMVAE plus LFSurv) assigns importance scores to input features and identifies the features that make important contribution in distinguishing between the high- and low-risk patients. The workflow of AUTOSurv is illustrated in Figure 6a.

### Data and preprocessing.

We collected survival outcomes (overall survival time and censoring status), demographic/clinical records (e.g., age, disease stage, race), and gene and miRNA expression data for 1,058 female patients with stage I - IV breast cancer, and for 355 female patients with stage I - IV ovarian cancer from the Genomic Data Commons (GDC) Breast Cancer (BRCA) cohort and Ovarian Cancer (OV) cohort of The Cancer Genome Atlas (TCGA) program, respectively. Data were downloaded from UCSC Xena data portal (https://xenabrowser.net/datapages/) [60] on October 30^th^, 2021. Demographics of the patients are summarized in Supplementary Table 1.1 and Supplementary Table 1.2. There were 175 and 222 observed deaths among the patients for the TCGA-BRCA dataset and TCGA-OV dataset, respectively. For both TCGA-BRCA and TCGA-OV datasets on the UCSC Xena portal, the gene expression data contain $log_{2}$-transformed fragments per kilobase of transcript per million mapped reads (FPKM) values, and the miRNA expression data contain $log_{2}$-transformed normalized counts in reads-per-million-miRNA-mapped (RPM).

We studied genes on autosomes and the X chromosome. For both BRCA and OV datasets, we randomly extracted 20% of patients as an external testing set which was not involved in any of the model training/tuning procedures during our experiments. The remaining 80% of patients were treated as a tuning set and further divided into training and validation sets with the ratio of 4:1. In each of the training/validation/testing sets, the gene/miRNA expression data were rescaled to the range of 0 to 1 using min-max normalization (Equation 1) to fit the input requirement of our VAE model [15]. To denoise the two omics data types, we followed the filtering procedure described in [61] and excluded genes/miRNAs with variance of < 0.02 in the min-max normalized tuning set. A summary of the omics features before and after preprocessing can be found in Supplementary Table 2. The min-max normalization process is summarized as follows:

(1) $$\nu_{minmax}^{\left(i\right)}\;=\;\frac{\nu^{\left(i\right)}-\nu_{min}}{\nu_{max}-\nu_{min}}$$

where $\nu^{\left(i\right)}$ and $\nu_{minmax}^{\left(i\right)}$ are the expression data values for feature ν in patient i before and after min-max normalization. $\nu_{max}(\nu_{min})$ is the maximum (minimum) value of ν across all patients in the dataset considered.

### Pathway-mask guided variational autoencoder.

We built a VAE model to compute lower-dimension latent variables $z^{\left(i\right)}\in \;{\mathbb{R}}^{d}$ from high-dimensional omics data ${\left\{x^{\left(i\right)}\right\}}_{i=1,\ldots ,N},x^{\left(i\right)}\in \;{\mathbb{R}}^{p}$ (N is the number of patients; d is the number of input features [e.g., number of genes]; d is the number of latent variables computed from the input data, and p ≫ d), which can reduce the risk of overfitting in the prognosis prediction task. Unlike classic autoencoder (AE) models, VAE learns a distribution estimate instead of a point estimate for the lower-dimension latent variables z [62], which can potentially increase the efficiency of the information extraction process and generate “disentangled” latent representations of the input features. This “disentanglement” allows qualitatively different information to be encoded into distinct latent variables, which could contribute to a more interpretable VAE model [63, 64].

As illustrated in Figure 6b, the encoder part of KL-PMVAE consists of a gene layer (each node represents a gene), a pathway layer (each node represents a pathway), and a miRNA layer (each node represents a miRNA). Reactome [65] pathway information was obtained from the online resource Database for Annotation, Visualization, and Integrated Discovery (DAVID) [66] (by the time we collected the pathway information, DAVID adopted Reactome database Version 78 in its knowledgebase [https://david.ncifcrf.gov/content.jsp?file=update.html]). DAVID allowed us to perform general or functional annotation for a long list of genes and compare results from different pathway databases all at once. We chose Reactome pathways because they have the widest coverage for the gene list we submitted. Sparse connections were forced between the gene layer and the pathway layer, where a gene node is connected to a pathway node only if that gene belongs to that specific pathway. According to [67], small pathways can be redundant with larger pathways and large pathways can be overly general, both of which can hamper the interpretability of the model. We can rephrase this in the sense of deep neural networks. On the one hand, pathway nodes that take information from too many gene nodes can complicate the interpretation process, especially when we are trying to identify the most important genes for model prediction. The pathway itself can be overly general from a biological perspective, and its relationship with disease outcome might be uninformative. Moreover, including “overly general” pathway nodes may result in too many trainable parameters in the deep neural network and make the model prone to overfitting. On the other hand, since we have thousands of genes in total, a pathway node connects to very few gene nodes may only encode trivial information, and detection of important features can be difficult when they are surrounded by noisy features. Therefore, we excluded pathways that contain fewer than 15 genes and pathways with more than 300 genes in our datasets. Note that there is no gold standard for deciding “too many” or “too few” gene-node connections for the pathway nodes; different selection criteria may result in varied model performance, but the comparison between different selection threshold is out of the scope of this study. Only genes belonging to at least one of the remaining pathways were kept. The same sparse connection pattern was kept between the last two layers in the gene part of the decoder (Figure 6b). This pathway-mask design is inspired by the mask-matrix-forced connections introduced by [6]. It not only incorporates prior biological knowledge into the network but also reduces the number of trainable parameters compared to a fully connected design and hence yields a lower risk of overfitting.

For multi-omics integration, there is a noticeable difference between the dimensionalities of the two omics data types (for both datasets, the number of genes is more than four times that of miRNAs, Supplementary Table 2). To mitigate potential imbalance in model training/parameter learning, we concatenated the pathway layer, instead of the gene layer, with the miRNA layer (Figure 6b). Because the pathway nodes contain forward-propagated-gene-node information, and we have 581 pathways which is comparable to the number of miRNAs. The concatenated features were then forward propagated to produce the means μ and log-variances $log\sigma^{2}$ for the latent variables $z|x∼N(\mu ,\;\sigma^{2})$, where $N(\mu ,\;\sigma^{2})$ is the estimated posterior distribution $q_{\phi }(z|x)$, ϕ is the set of learnable parameters in the encoder [15]. To sample from the distribution estimate, we applied the reparameterization trick:

(2) $$\hat{z}\;\;=\;\mu \;+\;\sigma \varepsilon ,\;\varepsilon ∼N\;(0,I)$$

which enables backpropagation for the VAE [62]. The decoder takes the sampled latent variable values $\hat{z}\;$ as input and reconstructs the gene expression and miRNA expression data (i.e., ${\hat{x}}_{gene}$ and ${\hat{x}}_{miRNA}$ respectively). The loss function for our VAE model is as follows:

(3) $$L_{VAE}\;=\;BCE(x_{gene},\;{\hat{x}}_{gene})\;+\;BCE(x_{miRNA},\;{\hat{x}}_{miRNA})\;+\;\beta D_{KL}(N(\mu ,\;\sigma^{2})||\;N\;(0,I))\;+\;\lambda_{1}{\left\|\theta_{1}\right\|}_{2}$$

where $BCE(x,\;\hat{x})$ is the binary cross-entropy between the input expression data x and the reconstructed expression data $\hat{x}$. The term $D_{KL}(N(\mu ,\;\sigma^{2})||\;N\;(0,I))$ is the KullbackLeibler (KL) divergence [68] between the estimated posterior distribution $N(\mu ,\;\sigma^{2})$ and the prior distribution N (0,I). The term ${\left\|\theta_{1}\right\|}_{2}$ is the 𝐿_2_-norm of the learnable parameters in KL-PMVAE and $\lambda_{1}$ is the regularization parameter that can be tuned to control the severity of the penalization. The value of β controls how much emphasis should be placed on the KL divergence term of the loss function and is set to 1 in conventional VAE but will be changed gradually from 0 to 1 in a KL-annealing learning scheme as described below. When KL divergence equals 0, the posterior equals an isotropic unit Gaussian regardless of the input features x; therefore, the minimization of the KL divergence term implies a limitation on the amount of information that can pass through the latent bottleneck (Figure 6b). According to [64], this constraint, combined with the pressure to minimize reconstruction loss, encourages the model to learn a more efficient representation of the data.

For model implementation, we applied batch normalization for all layers except for the latent bottleneck. Rectified linear units (ReLU), linear, and sigmoid activation functions were used for certain layers as illustrated in Figure 6b.

### Latent-feature-fed survival network for prognosis prediction.

To conduct survival analysis, we built a fully connected (FC) DL network as illustrated in Figure 6c, which can be viewed as a shallower version of DeepSurv [16]. The extracted latent features from KL-PMVAE (i.e., means μ of the learned distribution estimate of the latent variables z) were concatenated with the demographic/clinical variables (e.g., age, disease stage, race) and input into the network. After forward propagation through one hidden layer, the network outputs a prognostic index (PI) [6] for each patient, which is the estimate of the log-risk function in a CoxPH model [16]. High PI indicates a poor prognosis and vice versa. Like DeepSurv, the objective function of this FC network is the average negative log-partial likelihood with 𝐿_2_ regularization:

(4) $$l(\theta_{2})\;=\;-\frac{1}{n_{E=1}}\sum_{i:\;E_{i}=1}\left(PI_{i}\;-\;log\;\sum_{j\in R(T_{i})}e^{PI_{j}}\right)\;+\;\lambda_{2}{\left\|\theta_{2}\right\|}_{2}$$

where $n_{E=1}$ is the number of uncensored patients, and $R(T_{i})\;=\;\{i:\;T_{i}\;\geq \;t\}$ is the set of patients still at risk of failure at time t. The term ${\left\|\theta_{2}\right\|}_{2}$ is the 𝐿_2_-norm of the learnable parameters in LFSurv and $\lambda_{2}$ is the regularization parameter that can be tuned to control the severity of the penalization [6].

### Dropout was applied to prevent overfitting.

Hyperbolic tangent (tanh) activation was applied to compute node values for the hidden layer and linear activation was applied to compute the PI value in the output layer. Because the linear combination of predictors in CoxPH does not contain a constant term [17], the linear activation for the output layer has no bias term either.

### Prediction performance evaluation.

C-index was used to measure model performance in prognosis prediction: It counts concordant pairs between the predicted prognostic index and observed survival time [6, 69] and takes value between 0 and 1. C-index of 1 indicates perfect prediction and 0.5 is equivalent to random guessing.

### DeepSHAP for result interpretation.

DeepSHAP is an activation-based interpretation approach. According to [21], it avoids the saturation problem that perturbation- and gradient-based approaches fail to address. DeepSHAP shares the same key idea as DeepLIFT [21, 22]. The core assumption of DeepLIFT is the summation-to-delta property:

(5) $$\sum_{k=1}^{p\prime }C_{\Delta x_{k}\Delta t}\;=\;\Delta t$$

**Figure 7:**
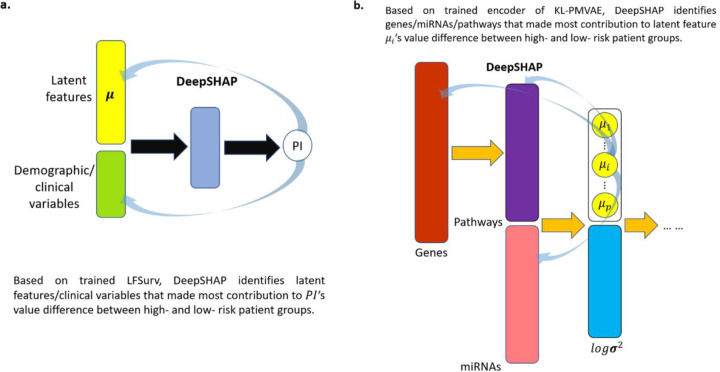
DeepSHAP implementations to identify (a) latent features/clinical variables that contribute most to the difference in PI between high- and low-risk groups, and (b) pathways/genes/miRNAs that contribute most to the difference in latent feature values (between high- and low-risk groups) for the most important latent features found in (a).

Here t represents some output neuron of interest, and $x_{1},\;x_{2},\;\ldots \;,x_{p\prime }$ represent some neurons in the input layer or an intermediate layer that are necessary and sufficient to compute t. Δt is the difference in output from some “reference” output, and $\Delta x_{k}$ is the difference in input from some “reference” input for $x_{k}$. $C_{\Delta x_{k}\Delta t}$ is the contribution score assigned to $\Delta x_{k}$ by DeepLIFT [21]. The summation-to-delta property hence states that the sum of the attributions over the input equals the difference-from-reference of the output [22]. Patients in the tuning set were assigned to the low-risk group if their predicted PI were smaller than the median $PI(PI_{med})$, and patients were assigned to the high-risk group if their predicted PI were higher than $PI_{med}$. During the interpretation procedures, we treated the low-risk group as the reference group. When backpropagating the predicted PI values in the trained LFSurv model via DeepSHAP, we can compute Shapley values (the contribution scores in this setting) for the latent features and clinical variables and identify the features that contribute most to the difference in PI between the low- and high-risk groups. Similarly, if we backpropagate the latent feature values in the trained KL-PMVAE model via DeepSHAP, we can identify which genes/miRNAs/pathways contribute most to the difference in latent feature values between the low- and high-risk groups. The +/− signs of the Shapley values imply the directions of the feature attributions, and higher absolute Shapley values correspond to greater contributions [20, 22]. Following the procedure proposed by [22], for each attempted DeepSHAP implementation, we randomly selected 100 samples from the low-risk group (which was our reference group) and 100 samples from the high-risk group, and for each feature its overall contribution was calculated by averaging its absolute Shapley values over the 100 high-risk group samples. The implementations of DeepSHAP are illustrated in Figure 7:

For objective (a) in Figure 7 (i.e., finding latent features/clinical variables that contributed most to the difference in PI between high- and low-risk groups), to make the comparison between different contribution scores more intuitive, we can divide the overall contribution score of each LFSurv input feature (i.e., latent feature or clinical variable) by the sum of overall contribution scores of all LFSurv input features and multiply by 100% (Equation 6). We denote this metric as “percent contribution”, since it reflects the amount of contribution from one specific feature compared to the total contribution from all input features.


(6) $$P_{i}\;=\;\frac{C_{i}}{\sum_{j=1}^{m}C_{j}}\;\times \;100\%$$

Assume that we have m variables in total (i.e., clinical variables plus latent features). Here $P_{i}$ is the percent contribution of variable *i*, which could be a clinical variable (e.g., age, clinical stage, race) or a latent feature (i.e., $\mu_{l}$), and $C_{i}$ is the overall contribution score for this variable.

### Model training/tuning and KL annealing.

As mentioned earlier, for both datasets, 20% of the whole data were kept as an external testing set that did not participate in any of the model training/tuning process. The remaining 80% were denoted as a tuning set and randomly divided into 80% training and 20% validation sets (64% and 16% of the whole data, respectively). Random division of the tuning set was carried out 10 times, which gave us 10 different training/validation sets. For each of the divisions, we trained the DNNs on the training set and conducted hyper-parameter tuning using the validation set. The set of hyper-parameters that gave the best model performance (i.e., lowest reconstruction loss for KL-PMVAE; or highest C-index for LFSurv) in the validation set were used to train the model on the whole tuning set. The trained model was then applied to the testing set to obtain the testing reconstruction loss/C-index. Ten different data divisions yielded 10 testing C-indices, and their mean and standard deviation were calculated. This scheme was applied on the same tuning set divisions and the testing set data when tuning/testing other models that we compared performance with. One-tailed Wilcoxon signed-rank test was applied to compare testing C-indices from different models or same models but in different cases. A p-value < 0.05 would suggest significant difference in prediction performance. The best sets of hyper-parameters (e.g., number of nodes in hidden layer, learning rate, regularization parameter 𝜆) were found via grid search. We summarized in Supplementary Table 3.1 and Supplementary Table 3.2 the lists of hyperparameters that we tuned.

We applied KL-annealing when training KL-PMVAE. During training, KL-annealing gradually increases β value from 0 to 1 in the loss function (Equation 3) and repeats this process for several cycles (the number of cycles and the cutting ratio in each cycle, illustrated in Supplementary Figure 1, were also tuned as hyperparameters) [59]. When β equals 0, the KL-divergence term has no influence on the loss function. The model learning is like a conventional autoencoder, which learns a point estimate for the latent variables. By gradually increasing β to 1 at the first part of each cycle and placing more weight on the KL-divergence term, $q_{\phi }\;(z|x)$ is regularized to change from learning a point estimate to learning a distribution estimate. For the rest of each cycle, β is fixed at value 1 to allow for optimizing the full VAE objective until convergence [59]. Because the learning process starts with random initialization, one key rationale behind KL-annealing is to prevent the distribution estimate from collapsing to the prior distribution (isotropic unit Gaussian N (0,I) in our case). In addition, according to the empirical results in [59], KL-annealing has the potential to increase reconstruction ability for VAE. By applying KL-annealing we expect to increase the efficiency of KL-PMVAE in information extraction.

## Supplementary Material

Supplement 1

## Figures and Tables

**Table 1:** Top 10 LFSurv input features for prognosis prediction

a. TCGA-BRCA		
Features	Overall contribution score	Percent contribution (%)
Age	0.592	19.60
Stage i	0.430	14.24
Stage ii	0.391	12.97
*μ*_5_	0.196	6.48
*μ*_2_	0.171	5.65
*μ*_10_	0.162	5.37
*μ*_8_	0.159	5.28
*μ*_1_	0.158	5.24
*μ*_9_	0.145	4.80
*μ*_13_	0.090	2.97
b. TCGA-OV		
Features	Overall contribution score	Percent contribution (%)
Age	0.464	38.74
Race	0.081	6.78
*μ*_16_	0.065	5.45
*μ*_3_	0.063	5.28
*μ*_4_	0.054	4.49
*μ*_10_	0.047	3.93
*μ*_12_	0.045	3.78
*μ*_6_	0.039	3.23
*μ*_9_	0.037	3.09
*μ*_7_	0.036	3.00

Percent contribution calculated according to Equation 6, as mentioned in the Methods section.
