# Supplementary material for: AUTOSURV: INTERPRETABLE DEEP LEARNING FRAMEWORK FOR CANCER SURVIVAL ANALYSIS INCORPORATING CLINICAL AND MULTI-OMICS DATA
Source: Res Sq. 2023 Aug 8:rs.3.rs-2486756. Preprint. [Version 1] doi: 10.21203/rs.3.rs-2486756/v1 (PMC10441464; doi:10.21203/rs.3.rs-2486756/v1)
Supplement: Supplement 1 [file NIHPPrs2486756v1-supplement-1.pdf]

Supplementary Files

This is a list of supplementary files associated with this preprint. Click to download.

- AUTOSurvsupplementarymaterials.docx
